# Pharmacological interventions for social cognitive impairments in schizophrenia: A systematic review and network meta-analysis of randomized controlled trials

**DOI:** 10.1192/j.eurpsy.2026.10159

**Published:** 2026-02-13

**Authors:** Yuji Yamada, Norio Watanabe, Yui Tomo, Hisateru Tachimori, Toru Horinouchi, Takashi Uchino, Ryotaro Kubota, Hiroki Okano, Shuhei Ishikawa, Ryo Sawagashira, Keisuke Takanobu, Yumi Hasegawa, Yohei Sasaki, Motohiro Nishiuchi, Tomiki Sumiyoshi, Naoki Hashimoto, Satoru Ikezawa, Takahiro Nemoto, Ryo Okubo

**Affiliations:** 1 Department of Forensic Psychiatry, National Center Hospital, National Center of Neurology and Psychiatry, Tokyo, Japan; 2 Department of Psychiatry, Soseikai General Hospital, Kyoto, Japan; 3 Clinical Research & Education Promotion Division, National Center of Neurology and Psychiatry, Tokyo, Japan; 4 Department of Health Policy and Management, Keio University School of Medicine, Tokyo, Japan; 5 Department of Information Medicine, National Institute of Neuroscience, National Center of Neurology and Psychiatry, Tokyo, Japan; 6 Department of Psychiatry, Hokkaido University Graduate School of Medicine, Hokkaido, Japan; 7 Department of Neuropsychiatry, Toho University Faculty of Medicine, Tokyo, Japan; 8 Department of Psychiatry, Fukushima Medical Center of Mental Health, Fukushima, Japan; 9 Forensic Psychiatry Center, Hokkaido University Hospital, Hokkaido, Japan; 10 Department of Preventive Intervention for Psychiatric Disorders, National Institute of Mental Health, National Center of Neurology and Psychiatry, Tokyo, Japan; 11 Faculty of Human Sciences, Musashino University, Tokyo, Japan; 12 Graduate School of Human and Social Sciences, Musashino University, Tokyo, Japan

**Keywords:** attributional bias, emotion perception, emotion processing, empathy, social cognition, social perception, theory of mind

## Abstract

**Background:**

Social cognitive impairments are a fundamental aspect of schizophrenia, exerting a substantial influence on patients’ functional outcomes. However, to date, there have been no meta-analyses of comprehensive pharmacological interventions covering all domains of social cognition. The aim of the present study was to address this knowledge gap by conducting a network meta-analysis, a comprehensive approach that systematically compares the efficacy of pharmacological interventions across all domains of social cognition.

**Methods:**

A literature search for randomized controlled trials (RCTs) was conducted using PubMed, Embase, the Cochrane Central Register of Controlled Trials, PsycINFO, ClinicalTrials.gov, and the International Clinical Trials Registry Platform. The Preferred Reporting Items for Systematic Reviews and Meta-Analysis guidelines were followed.

**Results:**

A total of 8,752 records were screened, and 60 RCTs involving 4,270 subjects were included in the systematic review. Thirty-six pharmacological interventions were extracted, but no compounds had a significant ameliorative effect on social cognition in comparison with placebo. In each domain of social cognition, the following compounds were identified as the most probable candidates for treatment selection: selective glycine uptake inhibitor (standardized mean difference [SMD], 0.46; 95% credible interval [CI], −0.52 to 1.44) and stimulant (SMD, 0.44; 95% CI, −0.57 to 1.45) for emotion perception in comparison with placebo. In the context of emotion processing, γ-aminobutyric acid (A) α2/α3 partial agonist (SMD, 0.33; 95% CI, −0.53 to 1.19) emerged as the top compound.

**Conclusions:**

To date, no pharmacological interventions have demonstrated efficacy for social cognitive impairments in schizophrenia.

## Introduction

Cognitive impairments represent an independent component of schizophrenia, exerting a substantial influence on the progression of the disease and having a pronounced impact on functional outcomes [1, 2]. Specifically, these factors encompass social isolation, stigma, substance use, poor dietary habits, and reduced prospects of finding a partner [1]. Furthermore, the unemployment rate of people with schizophrenia is reported to be more than 80% in Europe [3], making the effective treatment of schizophrenia an urgent issue. These conditions are largely attributable to impairments of neurocognition, including memory, attention, and executive functioning, as well as social cognition [4, 5]. Social cognition is defined as the cognitive processes underlying social interactions, and comprises the following domains: emotion perception (the ability to identify and recognize emotions from facial expressions or other cues), emotion processing (the ability to understand and manage emotions), theory of mind [ToM] (the ability to infer the mental states of others), social perception (the general processes required for decoding, understanding, and interpreting social cues), attributional bias (the way an individual usually interprets the cause of social behavior), and empathy [4, 5].

Social cognition plays a critical role in the social functioning of people with schizophrenia [5]. A meta-analysis revealed that people with schizophrenia exhibit significant impairments in the domains of emotion perception (Hedges’ *g* = 0.89), emotion processing (Hedges’ *g* = 0.88), ToM (Hedges’ *g* = 0.96), and social perception (Hedges’ *g* = 1.04) relative to healthy controls [6]. Furthermore, social cognition mediates the relationship between neurocognition and functional outcomes [7] and has a greater influence on real-world functioning than neurocognition [8]. Hence, the development of treatments for social cognitive impairments is an extremely important goal in schizophrenia.

Pharmacological interventions targeting social cognitive impairments have not been comprehensively evaluated in previous studies from two perspectives: intervention and outcome [9–13]. First, with regard to interventions, there have been individual meta-analyses on oxytocin [9], antipsychotics [10–12], and anti-dementia drugs [13], which indicates that intranasal oxytocin and atypical antipsychotic drugs may improve ToM [9] and emotion processing [10], respectively, while anti-dementia drugs (e.g., donepezil, galantamine, rivastigmine, and memantine) may elicit limited effects [13]; however, no network meta-analysis (NMA) comprehensively comparing the effects of interventions that include other drugs has been reported. Furthermore, NMA is also necessary when assessing randomized controlled trials (RCTs) that compare active drugs. Second, with respect to outcomes, the domains of social cognition were not considered [9, 13], or only the domains of ToM on oxytocin [9] and emotion processing on antipsychotics [10, 12] were included. To date, no comprehensive meta-analyses encompassing all domains of social cognition have been reported. Therefore, there is a strong need for further synthesis of existing research to facilitate the rational selection of compounds, with the aim of contributing to the development of novel therapeutic interventions to address social cognitive impairments.

To our knowledge, no quantitative comparison has been made regarding the ability of psychotropic drugs to enhance social cognition in people with schizophrenia. Notably, this is the first NMA to systematically and comprehensively examine the effects of a diverse array of pharmacological interventions on each domain of social cognition. This study signifies a substantial advancement in the development of treatments for social cognitive dysfunction.

## Methods

The Preferred Reporting Items for Systematic Reviews and Meta-Analysis (PRISMA 2020) guidelines were followed in the execution of this study [14]. The protocol was preregistered in the PROSPERO International Prospective Register of Systematic Reviews (registration number CRD42021293224), and a published protocol [15] is available.

### Search strategy

From inception to July 10, 2021, a comprehensive literature search was conducted using the following electronic bibliographic databases: PubMed, Embase, the Cochrane Central Register of Controlled Trials, PsycINFO, ClinicalTrials.gov, and the International Clinical Trials Registry Platform. The search terms were as follows: “schizophrenia,” “pharmacological intervention,” “social cognition,” and “randomized controlled trial” (see Supplementary 1Material 1) [15].

### Eligibility criteria

The following inclusion criteria [15] were applied to articles or clinical trial information:

1. RCTs including cluster and crossover trials.
2. Patients with schizophrenia or schizoaffective disorder based on F20–F29 of the International Statistical Classification of Diseases and Related Health Problems Version 10 or Schizophrenia Spectrum and Other Psychotic Disorders of the Diagnostic and Statistical Manual of Mental Disorders, 5th Edition (DSM-5), DSM-IV, or DSM-III.
3. Drug interventions (e.g., hormone-related drugs, psychostimulants, antidementia drugs, antipsychotic drugs, antibiotics, and supplements). With regard to the types of interventions, day-only or single doses were also included in this review.
4. At least one outcome was used for the evaluation of social cognition.

Original articles or clinical trial information written in any language were included.

### Study selection

YY removed duplicate studies before the initial screening. The 10 investigators (RK, HO, YH, YS, KT, SI, RS, TH, TU, and YY) were divided into five groups, with each group consisting of two investigators. The manuscripts were then divided into five batches by YY, with each pair of investigators overseeing one-fifth of all manuscripts. The first round of screening was conducted independently by each pair of investigators based on the original title and abstract. Subsequently, any study that was selected by at least one reviewer was subjected to a second round of screening. In the subsequent second screening, five groups of two reviewers each independently evaluated the eligibility of the full text. The senior reviewer (RO) was tasked with resolving any disagreements that arose in these evaluations [15].

### Data extraction

A total of six investigators (RK, HO, SI, TH, TU, and YY) were divided into three groups, with each group consisting of two individuals. These groups were tasked with independently extracting the following relevant information from the included studies: author, trial design, participants’ demographics, details of the intervention, and measurement tools. For measurements of social cognition, data were extracted through outcomes related to the following domains: emotion perception, emotion processing, ToM, social perception, attributional bias, and empathy [5]. Utilizing these data, we then proceeded to assess the effects of interventions on each of the six domains. The senior reviewer (RO) arbitrated in the event of any disagreements. In instances where the relevance of the measurement to social cognition was ambiguous, MN sought further elucidation from the authors regarding the outcomes. For ongoing clinical trial information included in the second screening, MN also requested detailed information from the researcher(s). In instances where MN did not receive a response from the author(s), they made up to three follow-up inquiries, separated by 1-week intervals. If this approach proved unsuccessful or impracticable, the studies in question were excluded from the analysis. It was noted that several studies satisfied the inclusion criteria for the systematic review but lacked pertinent information regarding the measures of social cognition that had been utilized. Consequently, the results of these studies were excluded from the meta-analysis [15].

### Quality assessments and risk of bias

Three groups of two investigators (RK, HO, SI, TH, TU, and YY) independently performed a quality assessment using the Cochrane Risk of Bias 2 Tool [16]. This tool assessed potential biases through signaling questions in the following domains: bias arising from the randomization process, bias due to deviations from intended interventions, bias due to missing outcome data, bias in measurement of the outcome, and bias in selection of the reported result. To enhance the precision of the quality assessments, all members participated in a training course on quality assessment using the Cochrane Risk of Bias 2 tool. This course included self-study materials and two days of exercises organized by Cochrane Japan. The senior reviewer (RO) arbitrated in the event of any disagreements [15].

### Targeted domains of social cognition and intervention

We focused on the following six domains of social cognition: emotion perception, emotion processing, ToM, social perception, attributional bias, and empathy [5]. For drug interventions, a wide variety of candidates were considered for inclusion (e.g., hormone-related drugs, psychostimulants, antidementia drugs, antipsychotic drugs, antidepressant drugs, antibiotics, and supplements) [15].

### Statistical analyses

For each domain of social cognition, we constructed a network diagram in which nodes represent the domains of the pharmacological interventions and edges connect interventions that were directly compared in the selected trials. Subsequently, a hierarchical Bayesian model was developed to obtain integrated estimates of the effect sizes between interventions. A detailed explanation regarding the model structure is provided in the Supplementary Materials (see Supplementary Material 2). The standardized mean difference (SMD) was used as the measure of observed effect sizes, and the SMD values in the selected trials were calculated using available information from each study. The specific definitions and calculation methods of the observed SMD are also described in the Supplementary Materials (Supplementary Material 2).

Posterior distributions of the effect sizes were sampled via Markov chain Monte Carlo (MCMC) simulation based on the Gibbs sampling algorithm. Four chains were run with a burn-in period of 5,000 iterations, followed by 100,000 iterations (thinned every 10th sample) per chain. Convergence was assessed by examining trace plots. If multi-arm trials were included in the analysis, inconsistency between direct and indirect comparison was examined using the node-splitting method, estimating the inconsistency factor, and comparing the analysis model with the unrelated mean effects model [17]. Heterogeneity between trials was evaluated via comparison of the analysis model with the unrelated study effects model. Model comparison was performed using the deviance information criterion [18]. More details and results on the inconsistency and heterogeneity evaluation are provided in the Supplementary 1Materials (see Supplementary Material 2, Figure S1, and Table S1). Sensitivity analyses were performed to assess the robustness of the results with respect to assumptions about the within-study correlation in multi-arm trials, detailed in the Supplementary Materials (Supplementary Material 2, Section 4). With regard to the assessment scales, sensitivity analyses were conducted using exclusively the scales recommended in the Social Cognition Psychometric Evaluation (SCOPE) study [5, 19]. In the context of the emotion processing domain, a sensitivity analysis was conducted employing solely the Mayer–Salovey–Caruso Emotional Intelligence Test (MSCEIT) [5]. Estimated effect sizes were presented as posterior means with 95% credible intervals (CIs), and the treatment rankograms were provided based on the cumulative probability of being ranked in terms of effectiveness among the interventions in the MCMC chains.

The analysis was conducted using R (ver. 4.2.2). Model construction and estimation were performed with the gemtc package [20]. We added a sampling function for the inconsistency factor to the original code in the package. The code with this addition is available from https://github.com/t-yui/gemtc/tree/inconsistency_factor.

## Results

**Figure 1.**
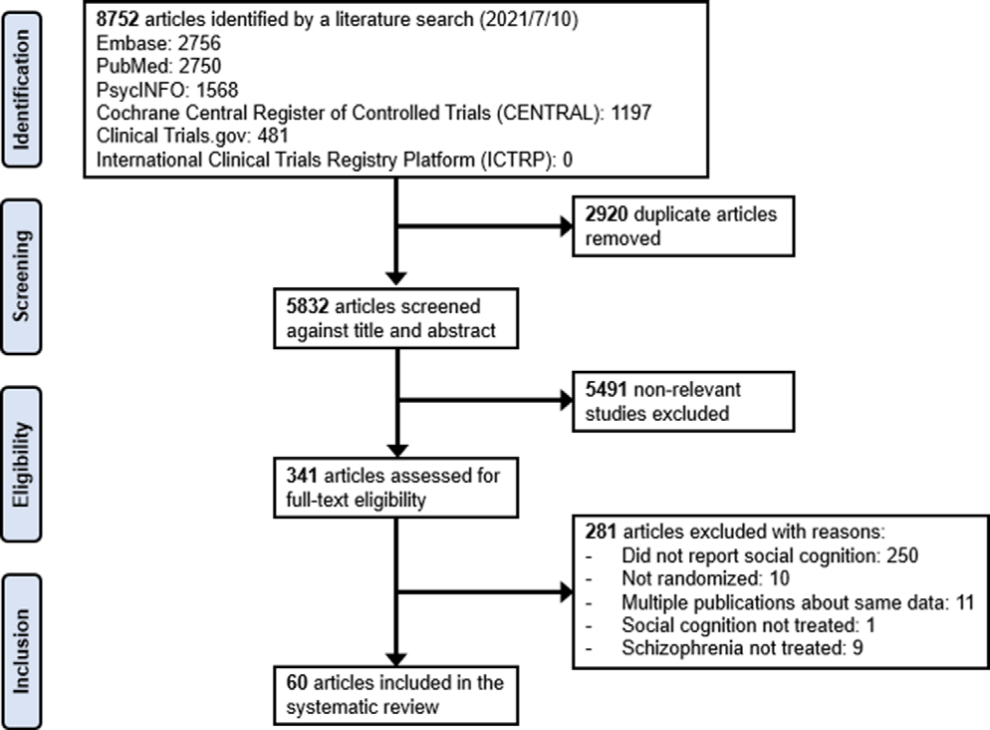
PRISMA study selection flowchart.

**Figure 2.**
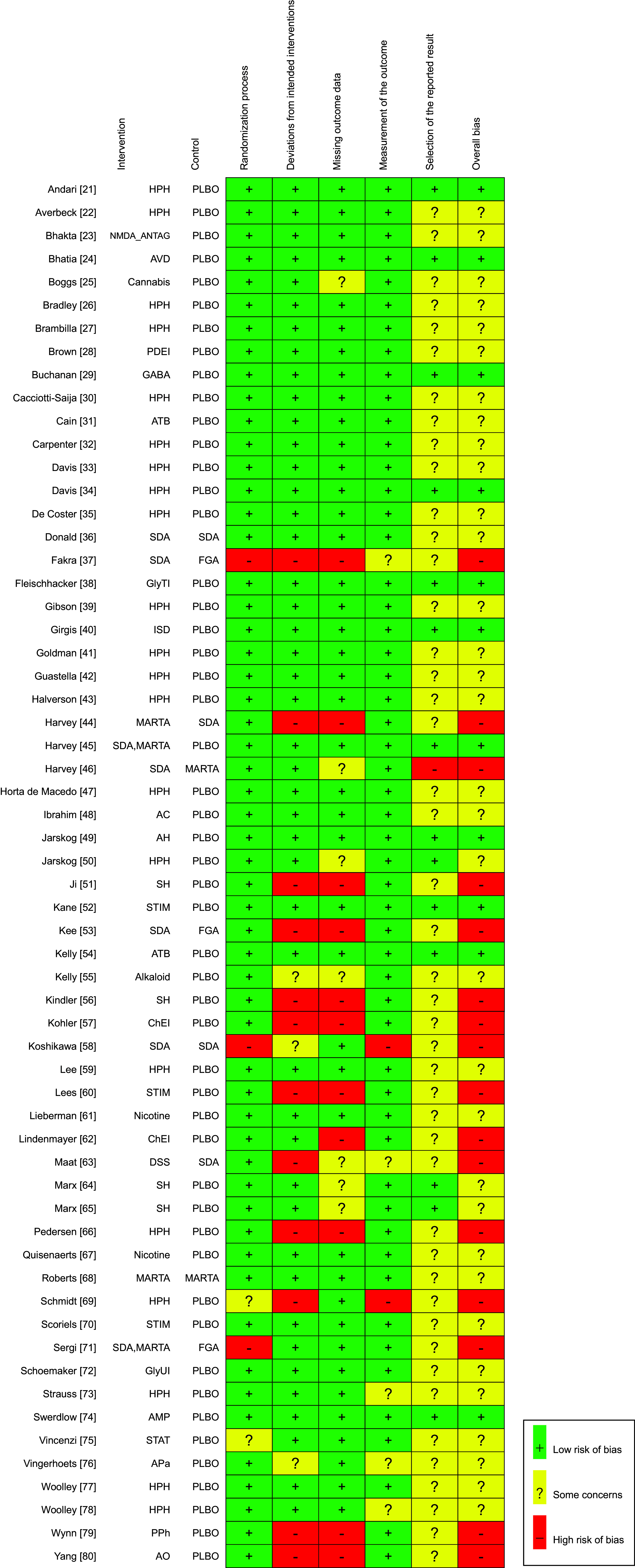
Risk of bias of included studies. HPH, hypothalamic hormone; NMDA_ANTAG, N-methyl-D-aspartate antagonist; AVD, antiviral drug; PDEI, phosphodiesterase 9 inhibitor; GABA, γ-aminobutyric acid (A) α2/α3 partial agonist; ATB, antibiotic; SDA, serotonin–dopamine antagonist; GlyTI, glycine transporter inhibitor; ISD, immunosuppressive drug; MARTA, multi-acting receptor-targeted antipsychotic; AC, anticonvulsant; AH, antihistamine; SH, sex hormone modulator; STIM, stimulant; ChEI, cholinesterase inhibitor; DSS, dopamine system stabilizer; GlyUI, selective glycine uptake inhibitor; AMP, amphetamine; STAT, statin; APa, antiparkinson; PPh, polyphenol; AO, antioxidant; PLBO, placebo; FGA, first-generation antipsychotic.

The preliminary search yielded a total of 8,752 records. After the elimination of duplicates, 5,832 articles were screened by title and abstract, leaving 341 potentially eligible records. A subsequent full-text review identified 60 articles that met the inclusion criteria for the systematic review. The primary reasons for exclusion were that the outcomes or the assessment of the outcomes did not align with the established inclusion criteria. The PRISMA study selection flowchart is shown in Figure 1. A summary of the risk of bias is presented in Figure 2.

Table 1 presents a comprehensive overview of the characteristics of the included studies. The 60 studies encompassed a total of 4,270 subjects. A total of 36 interventional agents were identified, which were subsequently classified into 27 domains based on their respective mechanisms of action (see Table 2). The hypothalamic hormone oxytocin emerged as the most prevalent intervention agent, with 21 studies (35% of all studies), followed by serotonin–dopamine antagonists with 7 studies (11%) (Tables 1 and 2). Regarding outcomes, rating scales were extracted for each domain of social cognition (Table 3). The majority of these domains were focused on emotion perception (31 studies, 51%), followed by emotion processing (24 studies, 40%) and ToM (15 studies, 25%). The MSCEIT was used for most of the emotion processing evaluations (22 studies), though it should be noted that multiple tools were employed, including the Penn Emotion Recognition Test (three studies) and Face Emotion Identification Test (two studies) in the domain of emotion perception, and the Reading the Mind in the Eyes Task (seven studies) and The Awareness of Social Inference Test (four studies) for the domain of ToM (see Tables 1 and 3). An evaluation of risk of bias was conducted. The results indicated that 55 (91.6% of all studies), 45 (75%), and 53 (88.3%) studies exhibited low risk of bias in the randomization process, deviations from intended interventions, and measurement of outcomes, respectively (Figure 2).

**Table 1.** Summary of included publications

	Study design		Intervention characteristics									Outcome domains
Reference	RCT type	Diagnosis	Medicines	Pharmacological actions/domains	Route	Dose per day	Control	Duration	Number of participants analyzed	Age(mean, SD) (intervention/control)	Female(%)	
Andari [21]	Parallel	SZ, SA	Oxytocin	HPH	Nasal	24 IU	Placebo	1D	20	50.8 (9.1)/ 47.7 (7.4)	0	Emotion perception Emotion processing
Averbeck [22]	Crossover	SZ	Oxytocin	HPH	Nasal	24 IU	Placebo	1D	21	38.2 (1.8)	0	Emotion perception
Bhakta [23]	Crossover	SZ, SA	Memantine	NMDA_ANTAG	Oral	20 mg	Placebo	1D	21	37.3 (7.2)	23.8	Emotion processing
Bhatia [24]	Parallel	SZ	Valacyclovir	AVD	Oral	3 g	Placebo	16 W	67	31.7 (8.5)/ 30.7 (8.6)	43.2	Emotion perception
Boggs [25]	Parallel	SZ	Cannabidiol	Cannabis	Oral	600 mg	Placebo	6 W	41	48.4 (9.3)/ 46.4 (9.5)	26.8	Emotion processing
Bradley [26]	Crossover	SZ	Oxytocin	HPH	Nasal	40 IU	Placebo	1D	26	42.7 (14.8)	100	Theory of mind
Brambilla [27]	Crossover	SZ	Oxytocin	HPH	Nasal	40 IU	Placebo	4 M	16	30.4 (6.7)	37.5	Theory of mind Emotion perception Emotion processing
Brown [28]	Parallel	SZ	BI 409306	PDEI	Oral	50 mg	Placebo	12 W	259	41.4 (9.5)/ 41.5 (9.7)	25	Emotion perception
Buchanan [29]	Parallel	SZ	MK-0777	GABA	Oral	8 mg	Placebo	4 W	42	44.9 (8.8)/ 40 (10.1)	30.9	Emotion processing
Cacciotti-Saija [30]	Parallel	SZ	Oxytocin	HPH	Nasal	48 IU	Placebo	6 W	52	21.5 (4.2)/ 22.3 (4.4)	30.7	Theory of mind Emotion perception Emotion processing
Cain [31]	Parallel	SZ	D-cycloserine	ATB	Oral	50 mg	Placebo	8 W	36	48.8 (11.5)/ 46.2 (13.3)	13.8	Emotion processing
Carpenter [32]	Parallel	SZ	Oxytocin	HPH	Nasal	48 IU	Placebo	4 W	38	47.4 (11.2)/ 42.2 (11.7)	13.8	Emotion processing
Davis [33]	Parallel	SZ	Oxytocin	HPH	Nasal	40 IU	Placebo	6 W	27	42.8 (9.1)/ 37 (10.8)	0	Theory of mind Social perception Emotion perception Emotion processing Empathy
Davis [34]	Parallel	SZ	Oxytocin	HPH	Nasal	40 IU	Placebo	1D	24	48.6 (6.6)/ 48.6 (9.1)	0	Theory of mind Social perception Emotion perception
De Coster [35]	Crossover	SZ	Oxytocin	HPH	Nasal	40 IU	Placebo	1D	27	35.3 (10.4)	0	Theory of mind
Donald [36]	Parallel	SZ	Ziprasidone	SDA	Oral	320 mg	Ziprasidone (160 mg)	8 W	75	39.2 (12)/ 41 (11.9)	30.6	Cognition (total)
Fakra [37]	Parallel	SZ	Risperidone	SDA	Oral	NA	Haloperidol	2 W	30	33.9 (9.5)/ 37.8 (11.4)	20	Emotion perception
Fleischhacker [38]	Parallel	SZ	BI 425809	GlyTI	Oral	10 mg	Placebo	12 W	255	37.9 (6.8)/ 37.2 (7.7)	32.9	Emotion processing
Gibson [39]	Parallel	SZ	Oxytocin	HPH	Nasal	48 IU	Placebo	6 W	34	38.8 (7.2)/ 35.6 (9)	8.8	Theory of mind Social perception Attributional bias Emotion perception
Girgis [40]	Parallel	SZ, SA	Tocilizumab	ISD	Intravenous	8 mg/kg	Placebo	12 W	36	43.2 (11.4)/ 41.6 (10.2)	30.5	Emotion processing
Goldman [41]	Crossover	SZ	Oxytocin	HPH	Nasal	20 IU	Placebo	1D	9	44 (9)	44.4	Emotion perception
Guastella [42]	Crossover	SZ	Oxytocin	HPH	Nasal	24 IU	Placebo	1D	13	37.4 (11.1)	0	Theory of mind Emotion perception
Halverson [43]	Parallel	SZ	Oxytocin	HPH	Nasal	48 IU	Placebo	12 W	68	41.4 (12.3)/ 35.9 (12.5)	22	Social perception
Harvey [44]	Parallel	SZ	Quetiapine	MARTA	Oral	200–800 mg	Risperidone (2–8 mg)	8 W	673	40.2 (10.5)/ 39.8 (10.8)	9.9	Emotion perception
Harvey [45]	Parallel	SZ	Lurasidone, Quetiapine	SDA, MARTA	Oral	80 mg, 600 mg	Placebo	6 W	361	37.8 (11.2), 37.3 (10.8)/ 37.1 (11)	34	Emotion perception
Harvey [46]	Parallel	SZ	Lurasidone	SDA	Oral	40–80 mg	Quetiapine (200–800 mg)	32 W	110	NA/ NA	NA	Emotion perception
Horta de Macedo [47]	Crossover	SZ	Oxytocin	HPH	Nasal	48 IU	Placebo	1D	20	29.6 (6.8)	0	Emotion perception
Ibrahim [48]	Parallel	SZ, SA	Valproate	AC	Oral	50–100 μg/mL	Placebo	16 W	94	25.2 (4.8)/ 26 (5.8)	60.6	Emotion perception
Jarskog [49]	Parallel	SZ	GSK239512	AH	Oral	20 μg, 40 μg	Placebo	7 W	50	38.2 (13.1)/ 40.1 (9.5)	20	Emotion perception Emotion processing
Jarskog [50]	Parallel	SZ, SA	Oxytocin	HPH	Nasal	48 IU	Placebo	12 W	68	41.9 (12.2)/ 36.1 (12.3)	22	Theory of mind Attributional bias Emotion perception
Ji [51]	Crossover	SZ, SA	Raloxifene	SH	Oral	120 mg	Placebo	6 W	20	36.5 (8.5)	40	Emotion perception
Kane [52]	Parallel	SZ	Armodafinil	STIM	Oral	200 mg	Placebo	4 W	30	41.4 (9.8)/ 46 (7.8)	23.3	Emotion processing
Kee [53]	Parallel	SZ	Risperidone	SDA	Oral	6 mg	Haloperidol (15 mg)	8 W	18	35 (9.7)/ 37.6 (8.3)	33.3	Emotion perception
Kelly [54]	Parallel	SZ, SA	Minocycline	ATB	Oral	200 mg	Placebo	10 W	52	42.9 (14.2)/ 42.3 (11)	25	Emotion processing
Kelly [55]	Parallel	SZ, SA	L-tetrahydropalmatine	Alkaloid	Oral	30 mg	Placebo	4 W	63	41.6 (11.1)/ 41.7 (10.1)	28.5	Emotion processing
Kindler [56]	Crossover	SZ, SA	Raloxifene	SH	Oral	120 mg	Placebo	6 W	30	35.5 (7.3)	26.6	Emotion processing
Kohler [57]	Parallel	SZ, SA	Donepezil	ChEI	Oral	≤10 mg	Placebo	16 W	26	31.7 (8)/ 30 (6.2)	23	Emotion perception
Koshikawa [58]	Parallel	SZ, SA	Risperidone LAI	SDA	Intramuscular	≤50 mg	Paliperidone palmitate (≤150 mg)	6 M	30	46.4 (10.3)/ 43.5 (11.7)	33.3	Emotion perception
Lee [59]	Parallel	SZ, SA	Oxytocin	HPH	Nasal	40 IU	Placebo	3 W	28	44.7 (11.7)/ 35 (8.2)	28.5	Theory of mind Social perception Attributional bias Emotion perception
Lees [60]	Crossover	SZ, SA	Modafinil	STIM	Oral	200 mg	Placebo	1D	46	25.6 (4.9)	21.7	Emotion processing
Lieberman [61]	Parallel	SZ	TC–5619	Nicotine	Oral	≤25 mg	Placebo	12 W	185	36.3 (NA)/ 36.3 (NA)	30.8	Emotion perception
Lindenmayer [62]	Parallel	SZ, SA	Galantamine	ChEI	Oral	≤24 mg	Placebo	28 W	38	41.9 (10.8)/ 38.5 (12.2)	26.3	Emotion perception
Maat [63]	Parallel	SZ	Aripiprazole	DSS	Oral	≤30 mg	Risperidone (≤6 mg)	8 W	80	26.4 (7.2)/ 24.8 (5)	11.2	Emotion perception Emotion processing
Marx [64]	Parallel	SZ, SA	Pregnenolone	SH	Oral	500 mg	Placebo	8 W	18	52.6 (6.3)/ 49.4 (12.1)	5.5	Emotion processing
Marx [65]	Parallel	SZ	Pregnenolone	SH	Oral	500 mg	Placebo	8 W	120	37 (8.4)/ 37.8 (8.4)	31.6	Emotion processing
Pedersen [66]	Parallel	SZ	Oxytocin	HPH	Nasal	48 IU	Placebo	2 W	20	39 (11.1)/ 35.7 (9.5)	15	Theory of mind
Quisenaerts [67]	Crossover	SZ	Nicotine	Nicotine	Oromucosal	1 mg	Placebo	1D	16	32.6 (8.8)	6.2	Theory of mind
Roberts [68]	Parallel	SZ	Olanzapine	MARTA	Oral	10–20 mg	Quetiapine (300–700 mg)	22 W	223	41.3 (9.4)/ 40.1 (9.3)	30.9	Social perception
Schmidt [69]	Crossover	SZ	Oxytocin	HPH	Nasal	40 IU	Placebo	1D	30	22.4 (4.6)	0	Theory of mind
Scoriels [70]	Crossover	SZ	Modafinil	STIM	Oral	200 mg	Placebo	1D	40	25 (2)	22.5	Emotion perception
Sergi [71]	Parallel	SZ	Risperidon, Olanzapine	SDA, MARTA	Oral	4 mg, 15 mg	Haloperidol (8 mg)	8 W	73	48.2 (7.7), 49.2 (6.7)/ 50 (5.8)	10.9	Social perception Emotion perception
Schoemaker [72]	Parallel	SZ	Org 25935	GlyUI	Oral	24–32 mg	Placebo	12 W	143	38.8 (11)/ 38.1 (10.5)	35.6	Emotion perception
Strauss [73]	Parallel	SZ	Oxytocin	HPH	Nasal	36 IU	Placebo	24 W	62	42.8 (8.7)/ 40.7 (10.2)	38.7	Theory of mind Social perception Emotion perception Empathy
Swerdlow [74]	Crossover	SZ	Amphetamine	AMP	Oral	10 mg	Placebo	1D	38	40.4 (7.1)	44.7	Emotion processing
Vincenzi [75]	Parallel	SZ	Pravastatin	STAT	Oral	40 mg	Placebo	12 W	60	42.5 (11)/ 44.5 (12.5)	36.6	Emotion processing
Vingerhoets [76]	Crossover	SZ, SA	Biperiden	APa	Oral	4 mg	Placebo	1D	33	27.6 (5.3)	33.3	Emotion processing
Woolley [77]	Crossover	SZ, SA	Oxytocin	HPH	Nasal	40 IU	Placebo	1D	29	44.6 (10.7)	0	Theory of mind
Woolley [78]	Crossover	SZ, SA	Oxytocin	HPH	Nasal	40 IU	Placebo	1D	33	42.9 (10.1)	9	Social perception
Wynn [79]	Parallel	SZ	Curcumin	PPh	Oral	360 mg	Placebo	8 W	39	50.1 (9.6)/ 50.9 (10.6)	15.3	Emotion processing
Yang [80]	Parallel	SZ, SA	N-acetylcysteine	AO	Oral	2400 mg	Placebo	8 W	34	NA/ NA	NA	Emotion processing

Abbreviations: RCT, randomized controlled trial; SD, standard deviation; D, day(s); W, week(s); M, month(s); SZ, schizophrenia; SA, schizoaffective disorder; NA, not available; LAI, long-acting injection; HPH, hypothalamic hormone; NMDA_ANTAG, N-methyl-D-aspartate antagonist; AVD, antiviral drug; PDEI, phosphodiesterase 9 inhibitor; GABA, γ-aminobutyric acid (A) α2/α3 partial agonist; ATB, antibiotic; SDA, serotonin–dopamine antagonist; GlyTI, glycine transporter inhibitor; ISD, immunosuppressive drug; MARTA, multi-acting receptor-targeted antipsychotic; AC, anticonvulsant; AH, antihistamine; SH, sex hormone modulator; STIM, stimulant; ChEI, cholinesterase inhibitor; DSS, dopamine system stabilizer; GlyUI, selective glycine uptake inhibitor; AMP, amphetamine; STAT, statin; APa, anti-Parkinson; PPh, polyphenol; AO, antioxidant.

**Table 2.** Pharmacological actions/domains

Medicines	Pharmacological actions/domains	Abbreviations
Oxytocin	Hypothalamic hormone	HPH
Haloperidol	First-generation antipsychotic	FGA
Lurasidone	Serotonin–dopamine antagonist	SDA
Paliperidone	Serotonin–dopamine antagonist	SDA
Ziprasidone	Serotonin–dopamine antagonist	SDA
Risperidone	Serotonin–dopamine antagonist	SDA
Quetiapine	Multi-acting receptor-targeted antipsychotic	MARTA
Olanzapine	Multi-acting receptor-targeted antipsychotic	MARTA
Aripiprazole	Dopamine system stabilizer	DSS
Raloxifene	Sex hormone modulator	SH
Pregnenolone	Sex hormone modulator	SH
Armodafinil	Stimulant	STIM
Modafinil	Stimulant	STIM
D-Cycloserine	Antibiotic	ATB
Minocycline	Antibiotic	ATB
Donepezil	Cholinesterase inhibitor	ChEI
Galantamine	Cholinesterase inhibitor	ChEI
Nicotine	Nicotine	Nicotine
TC-5619	Alpha-7 nicotinic receptor agonist	Nicotine
Valacyclovir	Antiviral drug	AVD
Tocilizumab	Immunosuppressive drug	ISD
N-Acetylcysteine	Antioxidant	AO
Valproate	Anticonvulsant	AC
GSK239512	Histamine H3 receptor antagonist (Antihistamine)	AH
Biperiden	Anti- Parkinson	APa
Amphetamine	Amphetamine	AMP
Cannabidiol	Cannabis	Cannabis
BI 409306	Phosphodiesterase 9 inhibitor	PDEI
BI 425809	Glycine Transporter inhibitor	GlyTI
Org 25935	Selective glycine uptake inhibitor	GlyUI
MK-0777	γ-Aminobutyric acid (A) α2/α3 partial agonist	GABA
Memantine	*N*-Methyl-D-aspartate antagonist	NMDA_ANTAG
L-Tetrahydropalmatine	Alkaloid	Alkaloid
Curcumin	Polyphenol	PPh
Pravastatin	Statin	STAT
Placebo	Placebo	PLBO

**Table 3.** Summary of the applied social cognition measures

	Outcome domains					
References	Theory of mind	Social perception	Attributional bias	Emotion perception	Emotion processing	Empathy/Others
Andari [21]				FEIT	MSCEIT	
Averbeck [22]				Hexagon Emotion Discrimination Task		
Bhakta [23]					MSCEIT	
Bhatia [24]				Penn-CNB		
Boggs [25]					MSCEIT	
Bradley [26]	TASIT					
Brambilla [27]	RMET			ERP	MSCEIT	
Brown [28]				CANTAB (emotion recognition)		
Buchanan [29]					MSCEIT	
Cacciotti-Saija [30]	RMET			FEEST		
Cain [31]					MSCEIT	
Carpenter [32]					MSCEIT	
Davis [33]	TASIT	PONS		Ekman Faces Tests	MSCEIT	EAT
Davis [34]	TASIT	Half-PONS		Ekman Faces Tests		
De Coster [35]	FBT					
Donald [36]						SCoRS (social cognition)
Fakra [37]				Feinberg Test		
Fleischhacker [38]					MSCEIT	
Gibson [39]	Eyes	Trustworthiness Task	AIHQ	ER40		
Girgis [40]					MSCEIT	
Goldman [41]				Ekman Faces Tests		
Guastella [42]	Hinting Task			Ekman Faces Tests		
Halverson [43]		IPT				
Harvey [44]				Penn Emotional Acuity Test		
Harvey [45]				CSSB (social cognition)		
Harvey [46]				CSSB (social cognition)		
Horta de Macedo [47]				Ekman Faces Tests		
Ibrahim [48]				ER40		
Jarskog [49]				CSSB (social cognition)	MSCEIT	
Jarskog [50]	RMET		AIHQ	ER40		
Ji [51]				Facial Emotion Recognition Task (angry accuracy)		
Kane [52]					MSCEIT	
Kee [53]				Facial Emotion Identification Test Voice Emotion Identification Test Videotape Affect Perception Test		
Kelly [54]					MSCEIT	
Kelly [55]					MSCEIT	
Kindler [56]					Emotional Go/No-Go task (accuracy)	
Kohler [57]				Emotion Discrimination and Differentiation Tests		
Koshikawa [58]				CSSB (social cognition)		
Lee [59]	RMET	Half-PONS	ASQ	FEIT		
Lees [60]					MSCEIT	
Lieberman [61]				CSSB (social cognition)		
Lindenmayer [62]				CSSB (social cognition)		
Maat [63]				Facial Affect Recognition Test	Emotional Working Memory Test	
Marx [64]					MSCEIT	
Marx [65]					MSCEIT	
Pedersen [66]	Brüne Theory of Mind Picture Stories Task					
Quisenaerts [67]	RMET					
Roberts [68]		SCRT				
Schmidt [69]	RMET					
Scoriels [70]				Emotion Recognition Task		
Sergi [71]		Half-PONS		Facial Emotion Identification Test		
Schoemaker [72]				Perception of Emotions Score		
Strauss [73]	RMET	Trust Game		FERT		EAT
Swerdlow [74]					MSCEIT	
Vincenzi [75]					MSCEIT	
Vingerhoets [76]					MSCEIT	
Woolley [77]	TASIT					
Woolley [78]		SJT				
Wynn [79]					MSCEIT	
Yang [80]					MSCEIT	

Abbreviations: TASIT, The Awareness of Social Inference Test; RMET, Reading the Mind in the Eyes Task; FBT, False Belief Task; Eyes, Reading the Mind in the Eyes Test; Half-PONS, Half Profile of Nonverbal Sensitivity; SCRT, Social Cue Recognition Task; IPT, Interpersonal Perception Task; SJT, Social Judgment Task; AIHQ, Ambiguous Intentions and Hostility Questionnaire; ASQ, Attributional Style Questionnaire; FEIT, Face Emotion Identification Test; Penn-CNB, Penn Cognitive Neuropsychological Battery; ERP, Emotional Priming Paradigm; FEEST, Facial Expressions of Emotions Task; ER40, Penn Emotion Recognition Test; CSSB, CogState Schizophrenia Battery; CANTAB, Cambridge Neuropsychological Test Automated Battery; FERT, Facial Emotion Recognition Test; MSCEIT, Mayer–Salovey–Caruso Emotional Intelligence Test; EAT, Empathic Accuracy Task; SCoRS, Schizophrenia Cognition Rating Scale.

**Figure 3.**
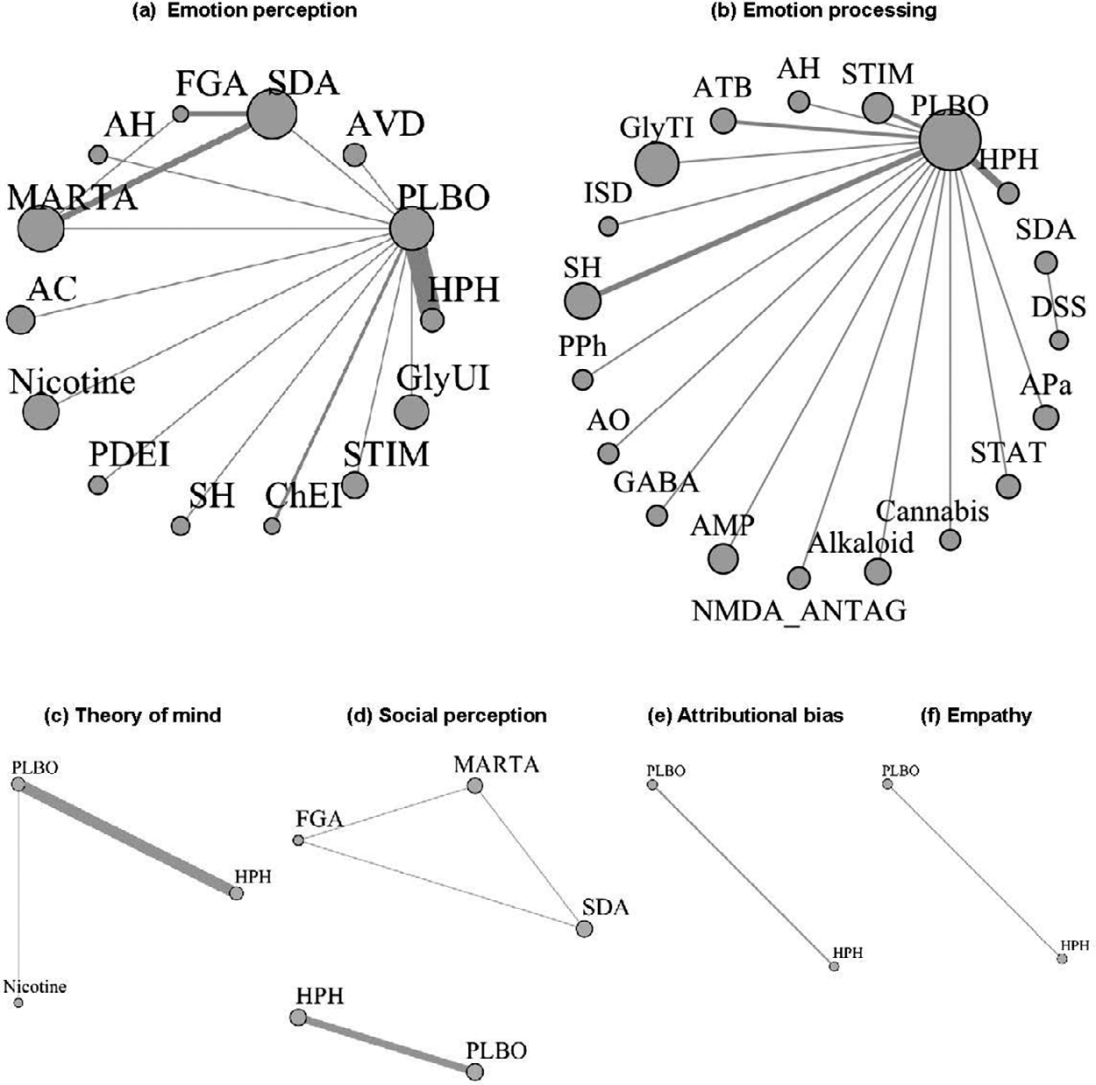
Network plot of outcomes related to social cognition. HPH, hypothalamic hormone; NMDA_ANTAG, N-methyl-D-aspartate antagonist; AVD, antiviral drug; PDEI, phosphodiesterase 9 inhibitor; GABA, γ-aminobutyric acid (A) α2/α3 partial agonist; ATB, antibiotic; SDA, serotonin–dopamine antagonist; GlyTI, glycine transporter inhibitor; ISD, immunosuppressive drug; MARTA, multi-acting receptor-targeted antipsychotic; AC, anticonvulsant; AH, antihistamine; SH, sex hormone modulator; STIM, stimulant; ChEI, cholinesterase inhibitor; DSS, dopamine system stabilizer; GlyUI, selective glycine uptake inhibitor; AMP, amphetamine; STAT, statin; APa, anti-Parkinson; PPh, polyphenol; AO, antioxidant; PLBO, placebo; FGA, first-generation antipsychotic.

The network plot for each domain of social cognition is shown in Figure 3. The thickness of the join line represents the number of studies, and the size of the node represents the number of subjects. In emotion perception, serotonin–dopamine antagonists, multi-acting receptor-targeted antipsychotics, and first-generation antipsychotics formed a closed loop, indirect comparison of multi-acting receptor-targeted antipsychotics versus first-generation antipsychotics (*p* = 0.2921, effect size, 0.89; 95% CI, −0.19 to 2.1). In emotion processing, the largest number of studies compared placebo and hypothalamic hormones, followed by sex hormone modulators. For ToM, the majority of the comparisons were between placebo and hypothalamic hormones. With respect to social perception, the network of serotonin–dopamine antagonists, multi-acting receptor-targeted antipsychotics, and first-generation antipsychotics formed a closed loop (Figure 3).

**Figure 4.**
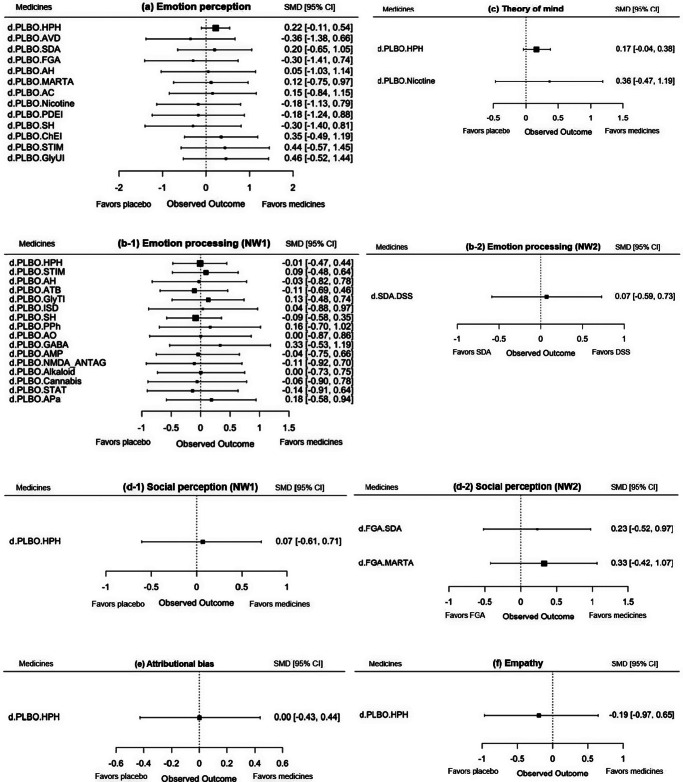
Forest plot of drugs versus placebo for outcomes related to social cognition. SMD, standardized mean difference; CI, credible interval; NW, network; HPH, hypothalamic hormone; NMDA_ANTAG, N-methyl-D-aspartate antagonist; AVD, antiviral drug; PDEI, phosphodiesterase 9 inhibitor; GABA, γ-aminobutyric acid (A) α2/α3 partial agonist; ATB, antibiotic; SDA, serotonin–dopamine antagonist; GlyTI, glycine transporter inhibitor; ISD, immunosuppressive drug; MARTA, multi-acting receptor-targeted antipsychotic; AC, anticonvulsant; AH, antihistamine; SH, sex hormone modulator; STIM, stimulant; ChEI, cholinesterase inhibitor; DSS, dopamine system stabilizer; GlyUI, selective glycine uptake inhibitor; AMP, amphetamine; STAT, statin; APa, anti-Parkinson; PPh, polyphenol; AO, antioxidant; PLBO, placebo; FGA, first-generation antipsychotic.

Forest plots of drugs (Figure 4) and league tables (Supplementary 1Figures 1–3) for each domain of social cognition are shown. The analysis revealed that no drug demonstrated a significant improvement in any domain with respect to emotion perception, as evidenced by the findings for hypothalamic hormones (SMD, 0.22; 95% CI, −0.11 to 0.54), selective glycine uptake inhibitors (SMD, 0.46; 95% CI, −0.52 to 1.44), and stimulants (SMD, 0.44; 95% CI, −0.57 to 1.45); for emotion processing, γ-aminobutyric acid (A) α2/α3 partial agonist (GABA), which was functionally selective for the α2 and α3 subunits, with 10–20% of the potency of a γ-aminobutyric acid (A) α2 full agonist (SMD, 0.33; 95% CI, −0.53 to 1.19); for ToM, hypothalamic hormones (SMD, 0.17; 95% CI, −0.04 to 0.38) and nicotine (SMD, 0.36; 95% CI, −0.47 to 1.19); and for social perception, hypothalamic hormones (SMD, 0.07; 95% CI, −0.61 to 0.71) (Figure 4). With regard to the assessment scales, sensitivity analyses were conducted using exclusively the scales recommended in the SCOPE study and using the MSCEIT (emotion processing domain) solely. However, the results were largely consistent with the main analysis (Supplementary 1Figure 4).

**Figure 5.**
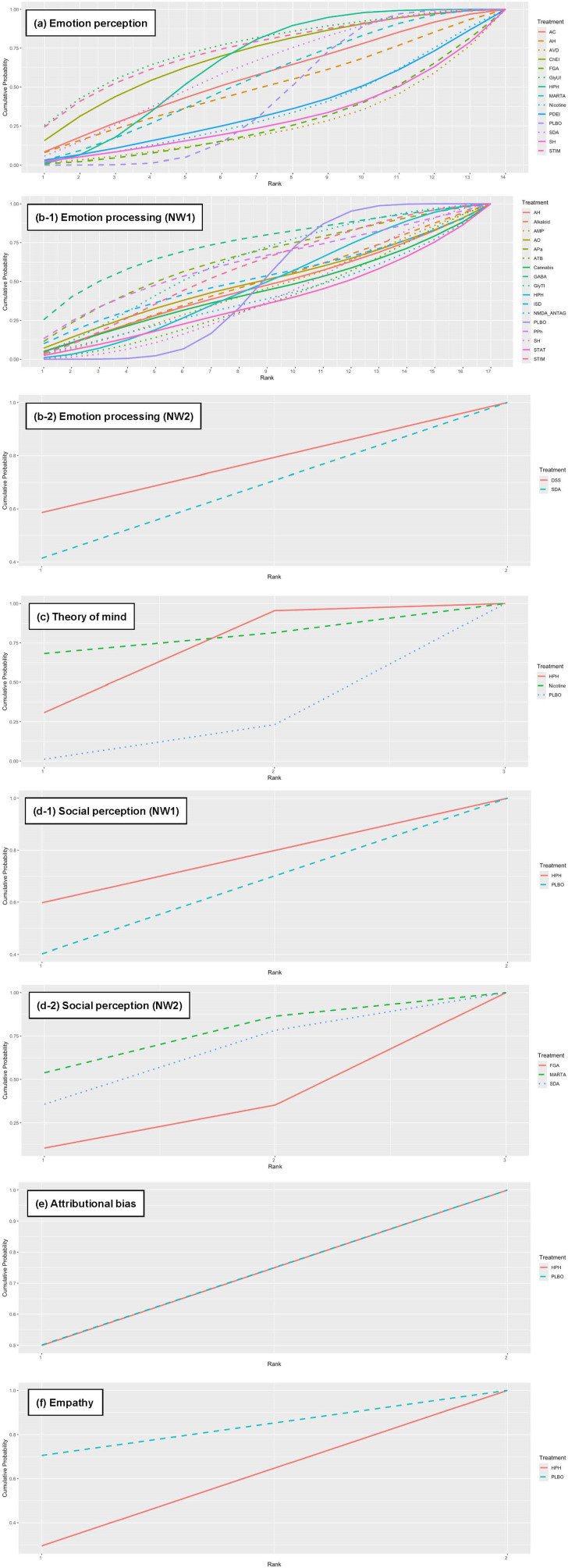
Ranked probability of outcomes related to social cognition. NW, network; HPH, hypothalamic hormone; NMDA_ANTAG, N-methyl-D-aspartate antagonist; AVD, antiviral drug; PDEI, phosphodiesterase 9 inhibitor; GABA, γ-aminobutyric acid (A) α2/α3 partial agonist; ATB, antibiotic; SDA, serotonin–dopamine antagonist; GlyTI, glycine transporter inhibitor; ISD, immunosuppressive drug; MARTA, multi-acting receptor-targeted antipsychotic; AC, anticonvulsant; AH, antihistamine; SH, sex hormone modulator; STIM, stimulant; ChEI, cholinesterase inhibitor; DSS, dopamine system stabilizer; GlyUI, selective glycine uptake inhibitor; AMP, amphetamine; STAT, statin; APa, anti-Parkinson; PPh, polyphenol; AO, antioxidant; PLBO, placebo; FGA, first-generation antipsychotic.

Figure 5 shows the rank probability of treatment choice for each domain of social cognition: for emotion perception, the most effective choice was selective glycine uptake inhibitors, followed by stimulants. For emotion processing, the most effective treatment was GABA. With respect to ToM, the best choice was nicotine (Figure 5).

## Discussion

In this study, we report the first NMA to comprehensively evaluate the effects of pharmacotherapy in all domains of social cognition. In the domains of ToM, social perception, attributional bias, and empathy, the number of studies was limited, and the networks were relatively sparse. A cautious approach is imperative when interpreting NMA in these domains, and it is anticipated that further accumulation of research findings will be necessary before a re-examination of these analyses can be undertaken. While no agents demonstrated substantial enhancement in each domain of social cognition, our findings identified several candidate compounds that offer directions for future research.

Oxytocin was the most frequently extracted intervention drug in this systematic review (35% of all studies), and no significant improvements were identified in any of the domains of social cognition. An earlier meta-analysis found an effect size of 0.2 when restricted to ToM, but no substantial improvement in overall social cognitive functioning [9]. Oxytocin, a neuropeptide predominantly synthesized in the hypothalamic nucleus, functions as a neurotransmitter and neuromodulator within the brain. Research has demonstrated its role in fostering social attachment and the formation of social bonds [81]. Therefore, it is assumed to be related to social cognition, and meta-analyses have reported improvement effects on empathy (Hedges’ *g* = 0.49) and ToM (Hedges’ *g* = 0.21) in neurodevelopmental disorders [82]. Conversely, the present review found that oxytocin did not enhance any domains of social cognition, indicating that the mechanism of action of oxytocin may differ in social cognition in neurodevelopmental disorders and social cognitive impairment in schizophrenia.

Glycine, stimulants, and GABA represent novel compounds that have emerged as promising candidates for the treatment of social cognitive impairments in schizophrenia. Specifically, selective glycine uptake inhibitors (SMD, 0.46) and stimulants (SMD, 0.44) have been identified as leading candidates for emotion perception, with GABA (SMD, 0.33) demonstrating promise in emotion processing (Figure 5). However, there are still a few validation studies of these candidate compounds, and the RCTs have been relatively small in size (Table 1). Further validation by large-scale RCTs is needed to determine the most effective therapeutic uses of these compounds.

Accurately measuring pharmacological effects on social cognition requires the implementation of uniform rating scales for each domain of social cognition. In the present study, we comprehensively examined the effects of drug intervention on domains of social cognition that had not been previously reported. However, our findings revealed no significant improvement in any domain. This may be indicative of genuine pharmacological ineffectiveness; however, methodological limitations, such as heterogeneity of tools, short treatment duration, and underpowered trials, are also considered to be involved. One factor contributing to this issue is the utilization of diverse scales for assessing social cognition, particularly in domains other than emotion processing (Table 3). This methodological approach poses a significant challenge in determining the effectiveness of these scales [83].

The development and dissemination of an international battery of measures of social cognition is an urgent issue. The SCOPE study sought to establish consensus on the theoretical structure of social cognition in schizophrenia and to systematically evaluate the psychometric properties of existing measures [19]. In the present study, the MSCEIT was utilized uniformly in 22 of 24 studies (91%) for the purpose of emotion processing. On the other hand, for the domains of emotion perception, ToM, social perception, and attributional bias, respectively, 12, 80, 11, and 66% of the studies employed the scales recommended in the SCOPE study. It is expected that, in the future, a comprehensive evaluation battery for social cognition, employing the recommended rating scales, will be developed and utilized in clinical trials.

In light of the pervasive utilization of pharmacological strategies for the treatment of schizophrenia, the current findings have substantial implications for enhancing social cognitive function. Notably, since no effective medication for social cognition currently exists, cognitive rehabilitation – which has demonstrated efficacy – remains the first-line treatment [5]. However, the identification of candidate compounds, such as selective glycine uptake inhibitors and GABA, suggests that future large-scale, targeted RCTs may reveal the potential to enhance social cognitive function.

## Limitations

This study has several limitations. First, the duration of illness, duration of medication, and concomitant therapy were not taken into account in the analysis. The intervention effects of various medications may differ depending on these factors. Especially, inclusion of very short-duration (e.g., 1-day) trials may dilute the capacity to detect meaningful effects. Second, given that no stratification is performed based on concomitant antipsychotic class, interactions with candidate drugs may occur. Third, there was little closed-loop formation in the current NMA. Therefore, indirect comparisons regarding the precise effects of various drugs could not be adequately examined. Finally, compounds with high rank probability of treatment choice were discussed as promising candidate drugs, but fundamentally, none of the drugs significantly improved social cognitive function compared to a placebo. The rank probability of treatment choice is merely an indicator of priority for each drug selection.

## Conclusions

At present, no pharmacological interventions have been demonstrated to be efficacious for social cognitive impairments in schizophrenia. Consequently, there is a need for future research to explore novel candidate compounds that diverge from conventional mechanisms of action and that are tailored to address social cognition. Moreover, the dissemination of a uniform test battery to measure social cognition is an urgent issue [5, 84]. In sum, a larger RCT is warranted to test the intervention effects of the new candidate compounds on each domain of social cognition.

## Supporting information

10.1192/j.eurpsy.2026.10159.sm001 Yamada et al. supplementary material Yamada et al. supplementary material

## Data Availability

Detailed search strategies and analysis methods are available online as Supplementary Materials.

## Supplementary material

The supplementary material for this article can be found at http://doi.org/10.1192/j.eurpsy.2026.10159.
