# Supplementary material for: Pharmacological interventions for social cognitive impairments in schizophrenia: A systematic review and network meta-analysis of randomized controlled trials
Source: Eur Psychiatry. 2026 Feb 13;69(1):e43. doi: 10.1192/j.eurpsy.2026.10159 (PMC13122521; doi:10.1192/j.eurpsy.2026.10159)
Supplement: Yamada et al. supplementary material [file S092493382610159Xsup001.zip › 20251106_Supplementary Figure 4..docx]

**
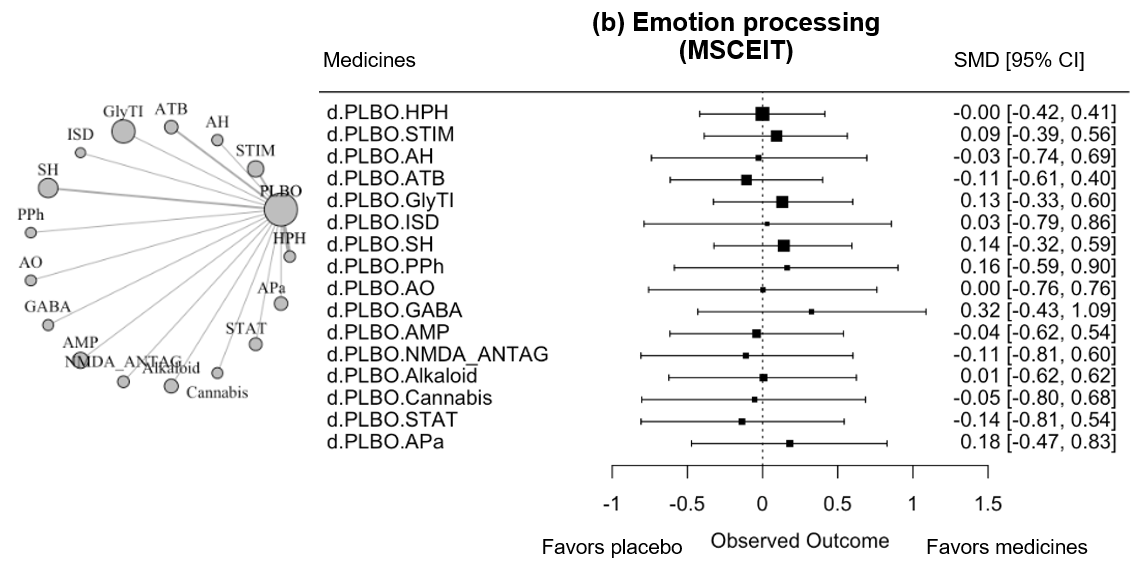

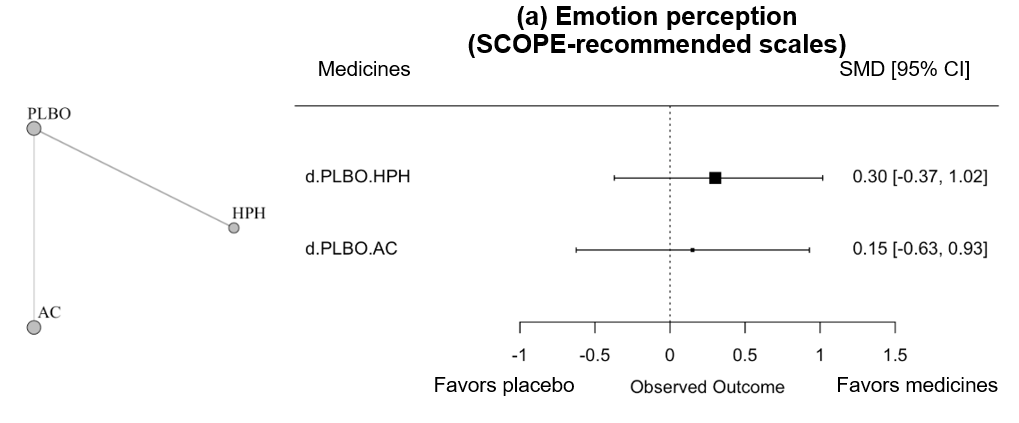
**

**
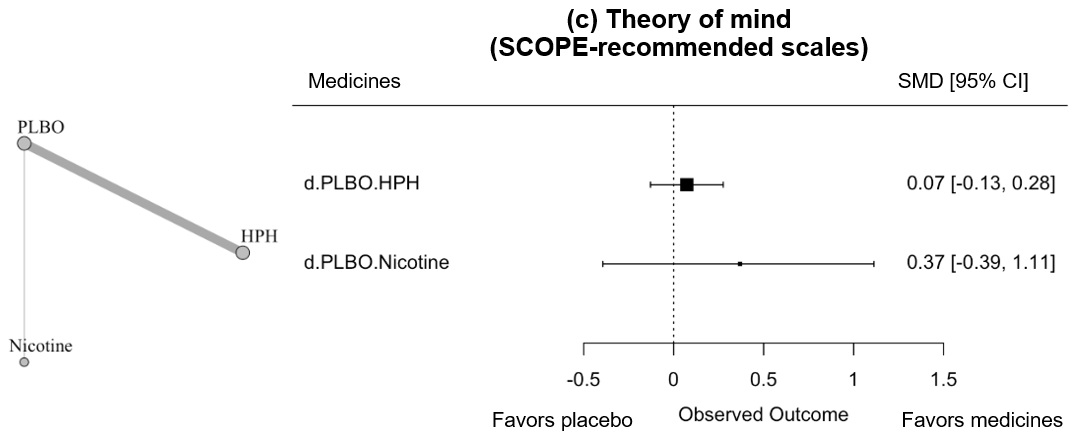
**

**
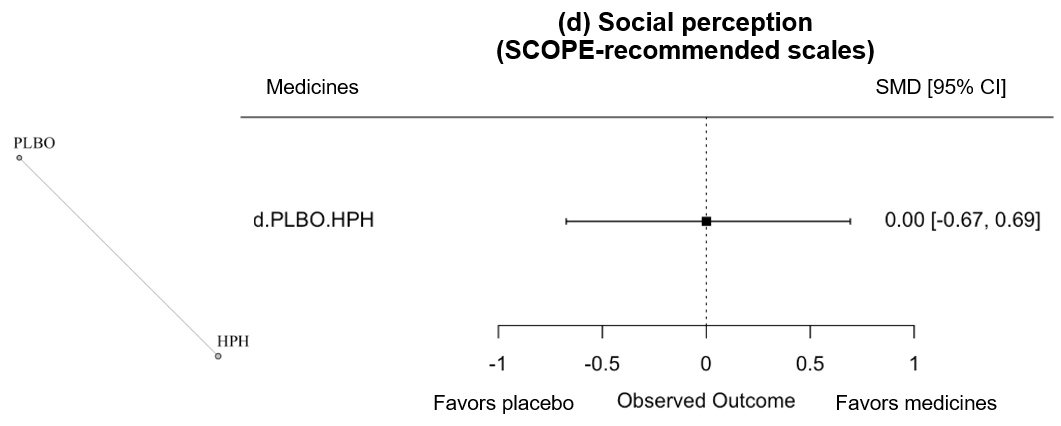
**

**
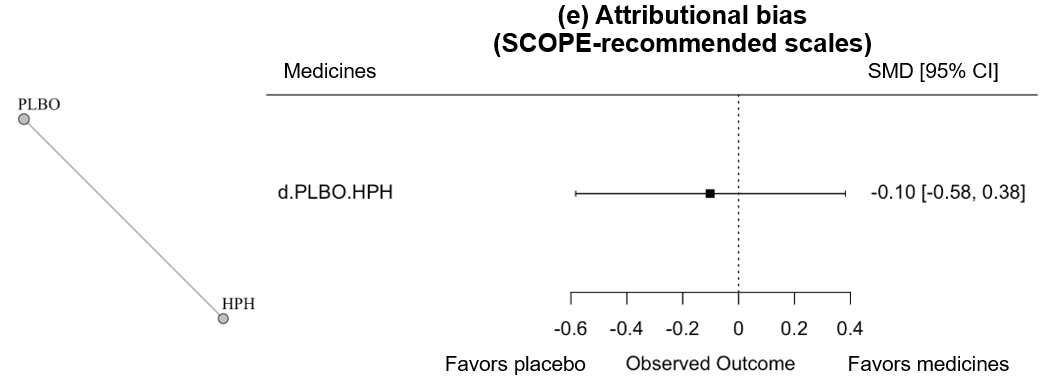
**

**Supplementary Figure 4. Network plot and Forest plot of drugs for outcomes about social cognition**

SMD, standardized mean difference; CI, credible interval; NW, network; HPH, hypothalamic hormone; NMDA_ANTAG, N-methyl-D-aspartate antagonist; AVD, antiviral drug; PDEI, phosphodiesterase 9 inhibitor; GABA, γ-aminobutyric acid (A) α2/α3 partial agonist; ATB, antibiotic; SDA, serotonin-dopamine antagonist; GlyTI, glycine transporter inhibitor; ISD, immunosuppressive drug; MARTA, multi-acting receptor-targeted antipsychotic; AC, anticonvulsant; AH, antihistamine; SH, sex hormone modulator; STIM, stimulant; ChEI, cholinesterase inhibitor; DSS, dopamine system stabilizer; GlyUI, selective glycine uptake inhibitor; AMP, amphetamine; STAT, statin; APa, antiparkinson; PPh, polyphenol; AO, antioxidant; PLBO, placebo; FGA, first-generation antipsychotic.
