# Supplementary material for: Pharmacological interventions for social cognitive impairments in schizophrenia: A systematic review and network meta-analysis of randomized controlled trials
Source: Eur Psychiatry. 2026 Feb 13;69(1):e43. doi: 10.1192/j.eurpsy.2026.10159 (PMC13122521; doi:10.1192/j.eurpsy.2026.10159)
Supplement: Yamada et al. supplementary material [file S092493382610159Xsup001.zip › 20250612_Supplementary Figure 3..docx]

**Theory of mind**

**
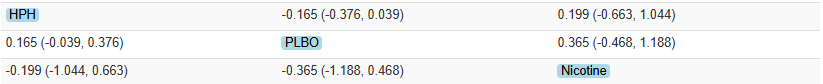
**

**Social perception (NW1)**

**
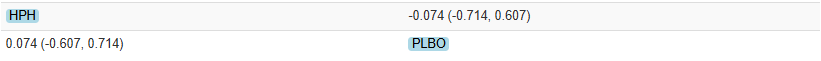
**

**Social perception (NW2)**

**
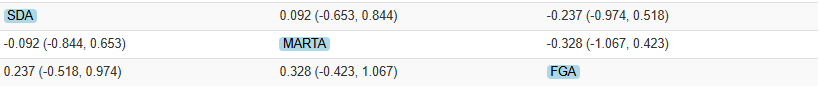
**

**Attributional bias**

**
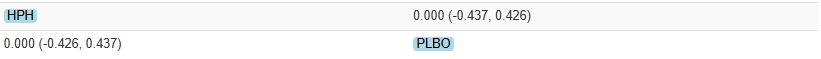
**

**Empathy**

**
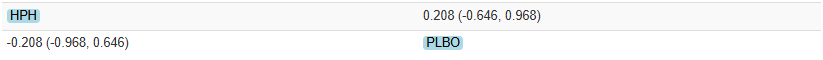
**

**Supplementary Figure 3. League table of outcomes related to theory of mind, social perception, attributional bias, and empathy**

Each cell represents the effect (in standardized mean difference with 95% credible interval) for the respective treatment comparison.

NW, network; HPH, hypothalamic hormone; SDA, serotonin-dopamine antagonist; MARTA, multi-acting receptor-targeted antipsychotic; PLBO, placebo; FGA, first-generation antipsychotic.
